# CASPR2 antibody-positive glioblastoma-associated pseudoparaneoplastic syndrome: a case report

**DOI:** 10.3389/fimmu.2026.1726790

**Published:** 2026-05-15

**Authors:** Huan Li, Yingjie Liu, Zhenqi Sun, Mingming Gu, Tian Zhan

**Affiliations:** 1 Department of Neurology, 970th Hospital of People's Liberation Army (PLA) Joint Logistic Support Force, Yantai, China; 2 Department of Radiology, 970th Hospital of People's Liberation Army (PLA) Joint Logistic Support Force, Yantai, China; 3 Department of Geriatrics, Shandong Provincial Third Hospital, Shandong University, Jinan, China

**Keywords:** autoimmune encephalitis, case report, CASPR2 antibody, glioblastoma, immunopathogenesis, neuroimmunology, neuro-oncology, paraneoplastic neurological syndrome

## Abstract

Paraneoplastic neurological syndromes (PNS) are rare immune-mediated disorders typically associated with systemic malignancies, while their occurrence in primary brain tumors is exceptionally uncommon. We report a 59-year-old man who presented with acute-onset epileptic seizures and transient serum CASPR2 antibody positivity, initially suggestive of autoimmune encephalitis. Early magnetic resonance imaging (MRI) showed T2-FLAIR hyperintensity in the right temporoparietal region without mass effect; however, follow-up imaging revealed a space-occupying lesion consistent with glioblastoma (GBM). The patient experienced transient improvement following immunoglobulin and corticosteroid therapy but achieved definitive recovery only after surgical tumor resection. Histopathology confirmed IDH1 wild-type GBM (CNS WHO Grade IV), and postoperative antibody seroconversion supported a diagnosis of pseudoparaneoplastic syndrome rather than true PNS. This case underscores that primary intracranial tumors such as GBM can transiently mimic autoimmune encephalitis through tumor-associated immune activation without direct antibody pathogenicity. Clinicians should maintain a high index of suspicion for tumor-related processes in patients with neuronal antibody positivity—particularly when antibodies are low-titer, serum-restricted, and clinically discordant—and prioritize dynamic imaging surveillance alongside serial antibody monitoring to avoid diagnostic delay. Further research is warranted to elucidate the mechanisms of GBM-induced immune dysregulation and its implications for neuroimmunological disease.

## Introduction

Paraneoplastic neurological syndromes (PNS) represent a group of neurological disorders mediated by immune mechanisms induced by tumors, rather than by direct metastasis or invasion. The classical mechanism underlying PNS is “molecular mimicry, “ in which immune responses directed against tumor antigens cross-react with neurons of the central or peripheral nervous system expressing homologous antigens, thereby resulting in neuronal dysfunction (1). These syndromes are most frequently associated with systemic malignancies, including lung, breast, and ovarian cancers, as well as thymoma. Characteristic autoantibodies directed against intracellular antigens—such as anti-Hu (ANNA-1), anti-Yo (PCA-1), and anti-Ri (ANNA-2)—can often be detected in the serum and cerebrospinal fluid (2).

In contrast, primary brain tumors—particularly glioblastoma (GBM)—have traditionally been regarded as infrequent causes of classic PNS, owing to their localization within the relatively “immunoprivileged” central nervous system (CNS) and their propensity to establish a profoundly immunosuppressive microenvironment (3). However, recent case reports have demonstrated that GBM may present with clinical and serological features that closely resemble autoimmune encephalitis (AE), including concomitant positivity for anti-NMDAR, anti-GABABR, or anti-Amphiphysin antibodies, thereby posing substantial diagnostic challenges (4, 5). Among these, cases associated with the presence of contactin-associated protein 2 (CASPR2) antibodies are exceedingly rare.

This report presents a case of GBM manifesting initially with epileptic seizures, accompanied by transient positivity for serum CASPR2 antibodies. The diagnostic and therapeutic course was complex, as initial imaging and serological findings led to a misleading diagnostic trajectory that was ultimately clarified through pathological confirmation. This case aims to elucidate the clinical characteristics, potential pathophysiological mechanisms, and management strategies of pseudoparaneoplastic syndromes associated with GBM, thereby enhancing clinicians’ awareness and improving their differential diagnostic capabilities for such rare entities.

## Clinical information

A 59-year-old man was admitted on September 24, 2024, due to a sudden loss of consciousness accompanied by generalized convulsions lasting approximately one hour. Approximately one hour prior to admission, while watching television at home, he experienced a sudden loss of consciousness with generalized tonic–clonic convulsions, jaw clenching, and frothing at the mouth. The convulsions subsided spontaneously after approximately one minute. Emergency medical services were contacted by family members, and the patient was subsequently transported to our hospital.

Neurological examination revealed that the patient was alert and oriented, with fluent speech and intact cranial nerves. Limb muscle strength was grade 5, tone was normal, and tendon reflexes were symmetrical. Deep and superficial sensations were preserved. Coordination and ataxia tests were normal, and bilateral pathological reflexes were absent. A preliminary diagnosis of epileptic seizure was made. Electroencephalographic (EEG) evaluation was performed. A routine EEG showed no obvious abnormalities. Long-term EEG monitoring demonstrated focal slow-wave activity in the right temporoparietal region, without interictal epileptiform discharges. There were no features suggestive of encephalitis or diffuse cortical dysfunction.

**Figure 1:**
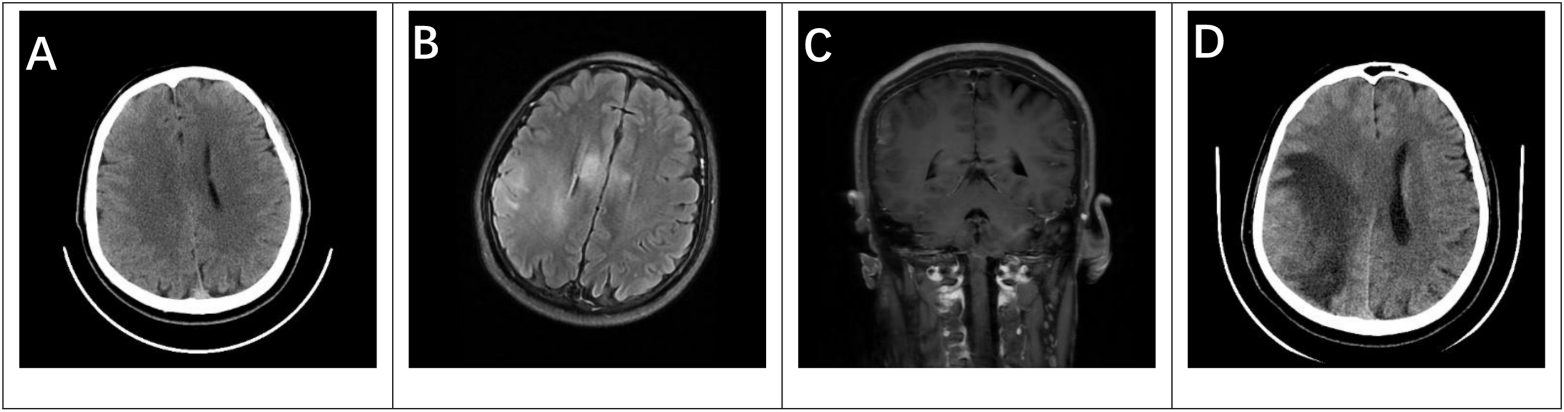
Patient’s cranial imaging findings from September 2024 **(A–C)** and February 2025 **(D)**. **(A)** Cranial CT shows no significant mass effect. **(B)** T2 Flair hyperintensity observed in the right temporal-parietal lobe and corpus callosum. **(C)** Contrast-enhanced cranial MRI shows enhancement in the right temporal-parietal meninges and parenchyma. **(D)** Cranial CT reveals a distinct mass effect.

Upon admission, cranial computed tomography (CT) revealed no significant mass effect (**Figure 1A**). Magnetic resonance imaging (MRI) demonstrated abnormal signal intensities in the right temporoparietal region and corpus callosum, with neoplastic lesions not excluded. Contrast-enhanced MRI suggested meningoencephalitis; however, tumor involvement could not be completely excluded. Further evaluation and follow-up imaging after treatment were recommended (**Figures 1B, C**). Routine laboratory tests, including blood biochemistry, complete blood count, coagulation profile, and D-dimer, were all within normal limits. Screening for eight common infectious disease markers yielded negative results. Serum tumor markers, including alpha-fetoprotein and carcinoembryonic antigen, were within normal limits. Cerebrospinal fluid (CSF) examination revealed an opening pressure of 170 mmH_2_O, normal routine parameters, and mildly elevated protein levels (477.9 mg/L). Given the combination of acute-onset seizure, abnormal MRI findings without a definite mass effect, and focal abnormalities on long-term EEG, autoimmune encephalitis was considered in the differential diagnosis. In addition, both serum and cerebrospinal fluid paraneoplastic antibody panels were negative. Based on this clinical context, autoimmune encephalitis-related antibodies were further tested. Autoimmune encephalitis antibody testing showed serum CASPR2 antibody positivity at a titer of 1:32 (cell-based assay), while CSF was negative for abnormal antibodies. Based on these findings, an initial working diagnosis of autoimmune encephalitis was made, and immunotherapy was initiated. Chest CT (128-slice) indicated pneumoconiosis with bilateral pleural thickening, and abdominal CT revealed hepatic cysts and cholelithiasis.

**Figure 2:**
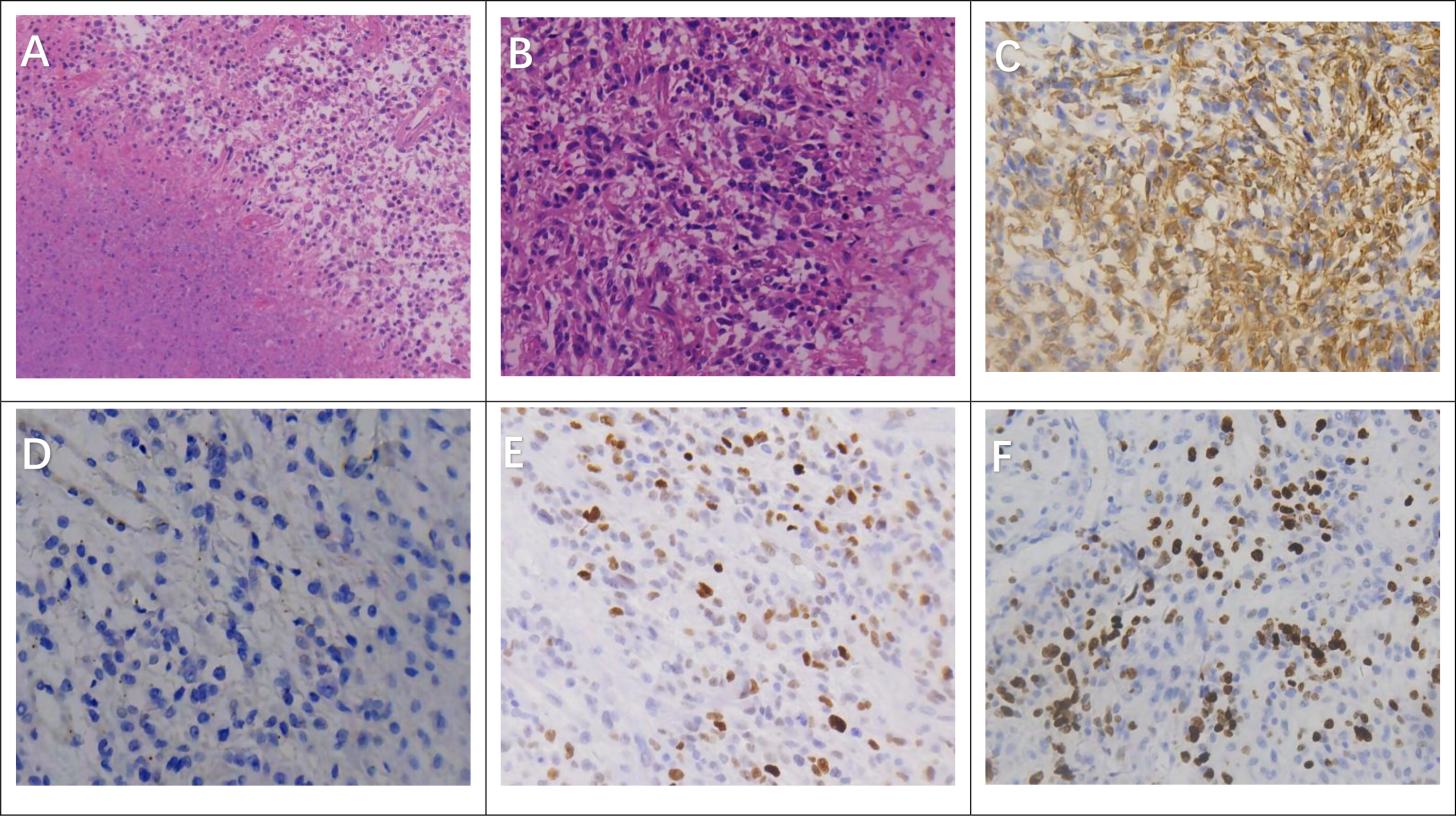
Surgical pathology findings in glioblastoma patients. **(A)** Extensive ischemic necrosis with pseudopalisading necrosis at the tumor periphery (H&E, ×20). **(B)** Tumors exhibit large, deeply stained nuclei with marked atypia (H&E, ×40). **(C)** Immunohistochemistry showing that most, but not all, tumor cells are positive for glial fibrillary acidic protein (GFAP, ×40). **(D)** Immunohistochemistry for IDH-1 demonstrating negative staining, consistent with wild-type status (IDH-1, ×40).**(E)** Nuclear accumulation of p53 in tumor cells, indicating mutant expression (p53, ×40). **(F)** High proliferative activity with a Ki-67 labeling index of approximately 35% (Ki-67, ×40).

**Figure 3:**
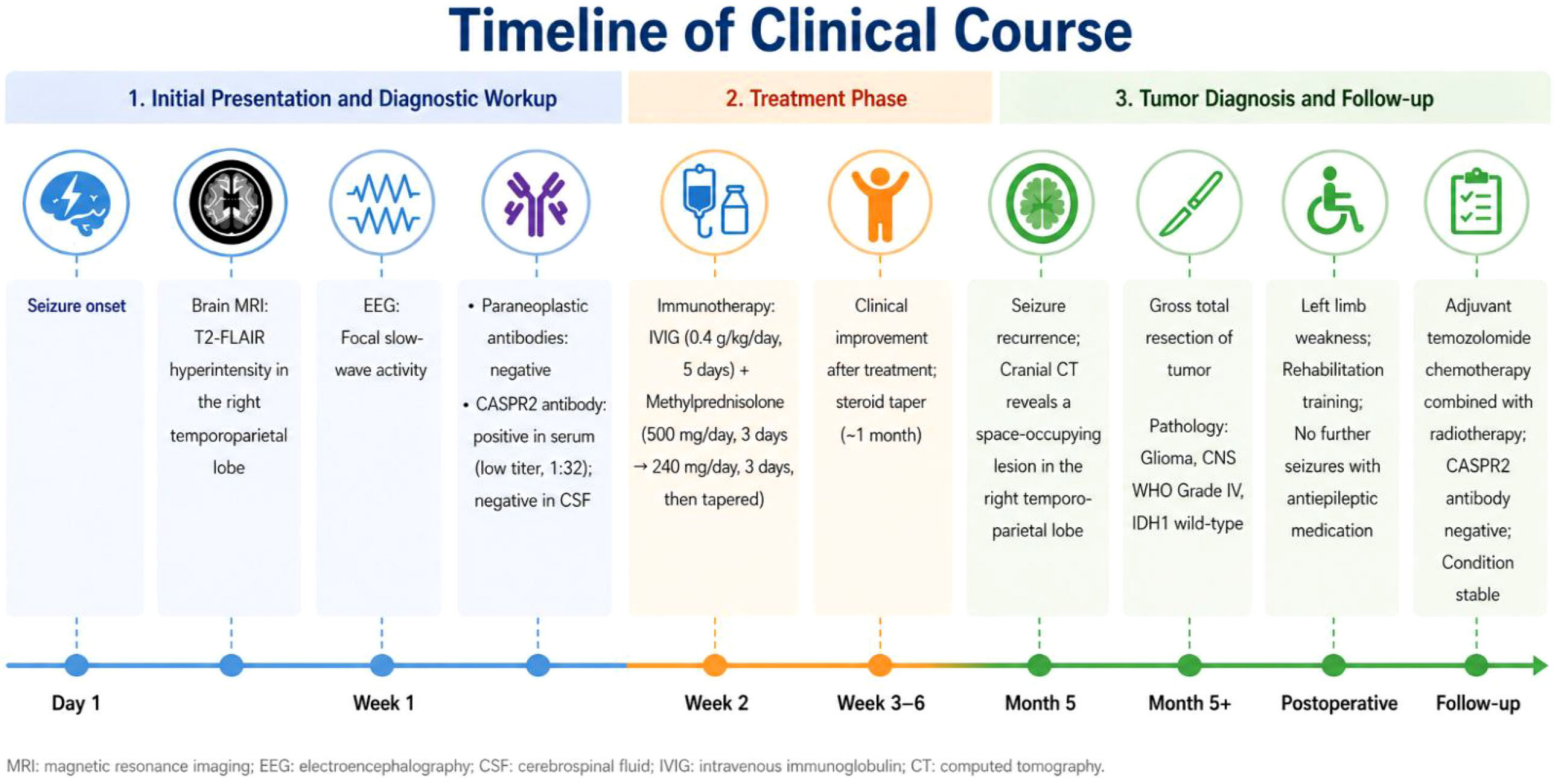
Timeline of the patient’s clinical course. The timeline summarizes the key events, including initial presentation with seizures, MRI and EEG findings, antibody testing, immunotherapy, recurrence of seizures, identification of a space-occupying lesion, surgical resection, adjuvant therapy, and follow-up outcomes.

The patient received intravenous immunoglobulin at a dose of 0.4 g/kg/day for 5 consecutive days. In addition, intravenous methylprednisolone was administered at 500 mg/day for 3 days, followed by 240 mg/day for another 3 days, and then gradually tapered to 60 mg/day. Subsequently, oral prednisone was initiated and reduced gradually (approximately 5 mg per week) until discontinuation. Following immunotherapy, the patient showed transient clinical improvement, with no immediate recurrence of seizures during the short-term period after treatment. On February 16, 2025, the patient experienced a recurrent seizure and was readmitted. Follow-up imaging at that time revealed a space-occupying lesion in the right temporoparietal region, indicating disease progression. Cranial CT revealed a mass lesion in the right temporoparietal lobe, suggestive of glioma (**Figure 1D**). The patient subsequently underwent gross total resection of the tumor, and the surgery was uneventful. Histopathological examination confirmed a right temporoparietal glioma, CNS WHO Grade IV, IDH1 wild-type (**Figure 2**). Postoperatively, the patient developed weakness of the left limbs. After rehabilitation training, he was gradually able to stand, although walking remained difficult. Oral antiepileptic medication was administered, and no further seizures occurred. The patient received regular adjuvant therapy with temozolomide combined with radiotherapy. At the three-month postoperative follow-up, serum autoimmune antibodies were negative, and during subsequent follow-up, the patient’s condition remained stable. A detailed timeline summarizing the clinical course, diagnostic workup, treatment, and follow-up is presented in **Figure 3**.

## Discussion

PNS are predominantly triggered by systemic malignancies and are characterized by relatively specific onconeural antibody profiles. For example, anti-Hu antibodies are most frequently associated with small cell lung cancer, anti-Yo antibodies with gynecologic malignancies, and anti-Ma2 antibodies with testicular germ cell tumors (2). As these antibodies target intracellular antigens, neuronal injury is largely mediated by cytotoxic T cells, leading to a poor prognosis and limited responsiveness to immunotherapy. Primary brain tumors, such as GBM, are rarely associated with PNS. This rarity may be attributable to the profoundly immunosuppressive tumor microenvironment of GBM, which hinders the development of effective systemic antitumor immune responses (3). In addition, limited antigen presentation within the central nervous system and restricted immune trafficking across the blood–brain barrier may further reduce the likelihood of detectable antineuronal antibodies. It is also possible that such antibodies are under-recognized, as neuronal antibody testing is not routinely performed in patients with primary CNS tumors. To date, only a few sporadic cases of GBM coexisting with autoimmune encephalitis–associated antibodies have been reported in the literature. Reported antibody types include anti-NMDAR, anti-GABABR, anti-amphiphysin, and anti–VGKC complex (untyped) antibodies (4, 5). To the best of our knowledge, no previous reports of CASPR2 antibody positivity in GBM have been identified in the existing literature despite systematic searches. CASPR2 is generally considered a low-risk neuronal surface antibody, with a weaker association with malignancy compared to classical onconeural antibodies such as amphiphysin. This case expands the current clinical spectrum and underscores the necessity of including primary intracranial tumors in the differential diagnosis when neuronal antibody positivity is detected, even for rare antibody types.

From a pathophysiological perspective, this case aligns more consistent with a pseudoparaneoplastic syndrome than with classical PNS. The fundamental mechanism of true PNS involves a specific cross-immunological response mediated by molecular mimicry. In contrast, pseudoparaneoplastic syndromes are typically attributed to tumor-related effects—such as mass effect, infiltration, or tumor-associated immune dysregulation—without evidence of direct antibody-mediated pathogenicity (6, 7). The diagnostic criteria for pseudoparaneoplastic syndromes include the following (1): antibodies are usually low-titer and restricted to serum, with no evidence of intrathecal synthesis (CSF-negative) (2); clinical manifestations correlate closely with the tumor location and associated mass effect (3); partial response to immunotherapy may occur due to the reduction of inflammatory edema, although definitive improvement depends on antitumor therapy; and (4) transient antibody positivity. In this case, the low-titer (1:32) serum CASPR2 antibody positivity with negative CSF findings aligns well with a ‘bystander’ effect, in which antibodies act as markers of tumor-associated immune activation rather than as direct pathogenic drivers. The occurrence of epileptic seizures corresponded closely to the right temporoparietal lesion, further supporting that the symptoms were predominantly tumor-induced. Moreover, the disappearance of serum antibodies following tumor resection indicates that their production was closely linked to tumor presence, consistent with the transient nature of pseudoparaneoplastic syndromes. Such antibodies typically resolve after tumor removal and do not indicate a persistent autoimmune state.

From a neuroimaging perspective, differentiating true PNS from pseudoparaneoplastic or pseudotumor syndromes is of considerable diagnostic importance. Typical MRI features of true autoimmune encephalitis (e.g., anti-LGI1 or anti-NMDAR encephalitis) include T2-FLAIR hyperintensity involving the limbic system—particularly the hippocampus and amygdala—usually bilateral, with minimal or absent parenchymal enhancement, occasional mild meningeal enhancement, and no significant mass effect (8). In contrast, when GBM mimics encephalitis, early MRI may demonstrate only nonspecific hyperintensity, whereas subsequent follow-up typically reveals rapid progression to characteristic tumor features, including prominent mass effect, abnormal contrast enhancement, and metabolic abnormalities on magnetic resonance spectroscopy (MRS) (9). In this case, the initial MRI report noted that “a tumor could not be entirely excluded, “ and a follow-up CT scan performed five months later revealed a space-occupying lesion consistent with progressive GBM. This dynamic radiological evolution underscores the necessity of short-interval imaging follow-up in cases with suspected autoimmune encephalitis but atypical features. Such an approach may help prevent delayed diagnosis of underlying tumors. In this context, the coexistence of low-titer serum CASPR2 positivity and nonspecific early imaging findings created a significant diagnostic challenge. At the initial stage, the presence of seizures, focal MRI abnormalities, and antibody positivity reasonably led to consideration of autoimmune encephalitis. However, the absence of typical CASPR2-associated clinical features and the subsequent evolution into a space-occupying lesion underscore the need for cautious interpretation of low-risk neuronal antibodies within the overall clinical and radiological context.

From a therapeutic perspective, the management of true PNS generally involves a combined approach integrating immunotherapy with definitive antitumor treatment (2, 10).In contrast, the cornerstone of pseudoparaneoplastic syndrome management is effective treatment of the underlying tumor. Although transient symptomatic improvement was observed following intravenous immunoglobulin and corticosteroid therapy, definitive resolution was achieved only after surgical tumor resection. Postoperative seroconversion further supported the pivotal role of antitumor therapy in disease control. In such cases, immunotherapy may primarily attenuate nonspecific inflammatory responses. However, excessive reliance on immunotherapy that delays surgical or chemoradiotherapeutic intervention may facilitate tumor progression and ultimately compromise prognosis. Therefore, accurate differentiation between true PNS and pseudoparaneoplastic syndromes is essential, as it directly influences therapeutic prioritization and patient outcomes.

The patient reported that the initial seizure occurred abruptly and caused substantial distress for both himself and his family. During the early phase of treatment, he experienced transient symptomatic relief following immunotherapy, which enhanced his confidence in the therapeutic approach. However, the subsequent recurrence of seizures and the eventual diagnosis of a brain tumor led to significant psychological burden. Following surgical intervention, the patient developed left-sided limb weakness; nevertheless, he actively engaged in rehabilitation and gradually regained the ability to stand. At the most recent follow-up, he reported no further seizure episodes and considered his condition to be stable under ongoing treatment. He expressed appreciation for the medical care received and emphasized the importance of timely diagnosis and continuous follow-up.

In summary, this case describes a rare presentation of glioblastoma with transient CASPR2 antibody positivity mimicking autoimmune encephalitis, ultimately representing a pseudoparaneoplastic phenomenon. It highlights the diagnostic challenges posed by the coexistence of nonspecific neuroimaging findings and non-pathogenic neuronal antibody positivity. Clinicians should interpret such antibody findings with caution and consider underlying structural lesions, particularly when clinical and radiological features are atypical. Timely repeat imaging and integrated clinical assessment are essential to avoid diagnostic delay and to guide appropriate tumor-directed management.

## Data Availability

The original contributions presented in the study are included in the article/supplementary material. Further inquiries can be directed to the corresponding authors.
